# A 'short walk' is longer before radiotherapy than afterwards: a qualitative study questioning the baseline and follow-up design

**DOI:** 10.1186/1477-7525-8-69

**Published:** 2010-07-16

**Authors:** Elsbeth F Taminiau-Bloem, Florence J van Zuuren, Margot A Koeneman, Bruce D Rapkin, Mechteld RM Visser, Caro CE Koning, Mirjam AG Sprangers

**Affiliations:** 1 Department of Medical Psychology, Academic Medical Center, University of Amsterdam, Amsterdam, The Netherlands; 2 Department of Clinical Psychology, University of Amsterdam, Amsterdam, The Netherlands; 3 Department of Epidemiology and Population Health, Albert Einstein College of Medicine, New York, USA; 4 Department of General Practice, Academic Medical Center, University of Amsterdam, Amsterdam, The Netherlands; 5 Department of Radiotherapy, Academic Medical Center, University of Amsterdam, Amsterdam, The Netherlands

## Abstract

**Background:**

Numerous studies have indirectly demonstrated changes in the content of respondents' QoL appraisal process over time by revealing response-shift effects. This is the first known study to qualitatively examine the assumption of consistency in the content of the cognitive processes underlying QoL appraisal over time. Specific objectives are to examine whether the content of each distinct cognitive process underlying QoL appraisal is (dis)similar over time and whether patterns of (dis)similarity can be discerned across and within patients and/or items.

**Methods:**

We conducted cognitive think-aloud interviews with 50 cancer patients prior to and following radiotherapy to elicit cognitive processes underlying the assessment of 7 EORTC QLQ-C30 items. Qualitative analysis of patients' responses at baseline and follow-up was independently carried out by 2 researchers by means of an analysis scheme based on the cognitive process models of Tourangeau et al. and Rapkin & Schwartz.

**Results:**

The interviews yielded 342 comparisons of baseline and follow-up responses, which were analyzed according to the five cognitive processes underlying QoL appraisal. The content of comprehension/frame of reference changed in 188 comparisons; retrieval/sampling strategy in 246; standards of comparison in 152; judgment/combinatory algorithm in 113; and reporting and response selection in 141 comparisons. Overall, in 322 comparisons of responses (94%) the content of at least one cognitive component changed over time. We could not discern patterns of (dis)similarity since the content of each of the cognitive processes differed across and within patients and/or items. Additionally, differences found in the content of a cognitive process for one item was not found to influence dissimilarity in the content of that same cognitive process for the subsequent item.

**Conclusions:**

The assumption of consistency in the content of the cognitive processes underlying QoL appraisal over time was not found to be in line with the cognitive processes described by the respondents. Additionally, we could not discern patterns of (dis)similarity across and within patients and/or items. In building on cognitive process models and the response shift literature, this study contributes to a better understanding of patient-reported QoL appraisal over time.

## Background

Clinical research increasingly assesses change in quality of life (QoL) to demonstrate the effect of treatment beyond clinical efficacy and safety [1-3]. Additionally, change in QoL is assessed as part of cost utility evaluations and evaluations of psychological interventions [4]. The prospective baseline and follow-up design is most commonly used to assess change in QoL. The mean change in score from baseline to follow-up (i.e. paired difference) provides an indication of the amount and direction of change. This design implicitly assumes consistency in the content of respondents' QoL appraisal process over time. For example, respondents are assumed to refer to the same concept of the target construct over time. Changes in the content of respondents' QoL appraisal process may render QoL assessments over time incomparable.

Numerous studies have indirectly demonstrated that the content of respondents' QoL appraisal process changes over time by revealing response-shift effects [e.g. [5-9]]. However, direct evidence regarding such changes in QoL appraisal generated by the baseline and follow-up design is lacking, i.e. insight into the content of the cognitive processes underlying QoL appraisal over time. In this study, we will qualitatively examine the assumption of consistency in the content of the cognitive processes underlying QoL appraisal over time. To reflect the measurement of change in QoL in the context of clinical research, we will examine the content of patients' QoL appraisal process prior to and at the end of radiotherapy.

The cognitive processes underlying QoL assessment are described by Rapkin & Schwartz in their theoretical model of QoL appraisal [10]. This model distinguishes four cognitive processes; 1) induction of a frame of reference; 2) recall and sampling of salient experiences; 3) use of standards of comparison against which each sampled experience is judged; and 4) use of an algorithm to prioritize and combine all retrieved samples to arrive at a QoL score. Previously, Tourangeau et al. [11] had developed a cognitive process model in the area of survey research to describe the cognitive processes underlying responses to questionnaire items. This model shows great resemblance to the Rapkin & Schwartz model, as it encompasses a) comprehension and interpretation of the question; b) recall of relevant information; and c) combination of the retrieved information. The Tourangeau model does not include the use of standards of comparison, but adds the cognitive component d) reporting and response selection, according to which the respondent may edit the initial response and subsequently maps the judgment onto the appropriate response category. Combined, the models of Tourangeau et al. [11] and Rapkin & Schwartz [10] thus entail five cognitive processes (see Table 1).

**Table 1:** Cognitive process models of Tourangeau et al. and Rapkin & Schwartz

**Tourangeau et al**. - survey answering model -	Rapkin & Schwartz - QoL appraisal model -	Example interview probes
Comprehension	Frame of reference	What does [target construct in item, e.g. quality of life] mean to you?

Retrieval	Sampling strategy	Can you tell me how you came to think of [aspect mentioned by respondent]?

	Standards of comparison	Did you compare yourself to someone or something?

Judgment	Combinatory algorithm	How did you arrive at your response?

Reporting and response selection		Can you tell me why you choose the selected response category?

A number of studies [e.g. [12-15]] qualitatively investigated the content of the first cognitive process of the models of Tourangeau et al. [11] and Rapkin & Schwartz [10], i.e. comprehension and frame of reference respectively. These studies thus focused solely on possible changes in patients' definition of the concept QoL over time. To the best of our knowledge, the present study is the first to qualitatively examine whether cancer patients' QoL appraisal processes remain similar or rather change over time by examining the content of all five cognitive processes underlying QoL evaluation. To that end, we have combined both models in a qualitative analysis scheme, which proved applicable in the qualitative analysis of the cognitive processes underlying responses to QoL items [16]. The study's specific objectives are to examine whether the content of each distinct cognitive process underlying QoL appraisal is (dis)similar over time and whether patterns of (dis)similarity can be discerned across and within patients and/or items.

## Methods

### Participants

The study sample comprised cancer patients undergoing treatment at the Department of Radiotherapy of the Academic Medical Center (AMC) in Amsterdam fulfilling the following inclusion criteria: a minimum age of 18 years, fluent command of Dutch, absence of cognitive impairments, not diagnosed with a brain tumor and/or treated with brain irradiation, expected survival of at least 3 months, and undergoing a minimum radiotherapeutic treatment of 3 weeks. Two researchers (ETB, MK) further selected newly diagnosed cancer patients purposively according to patient characteristics (i.e. gender, age, tumor site, and length of radiotherapeutic treatment) to ensure a heterogeneous sample and wide variation in cognitive processes used. Radiotherapists recruited these selected patients and provided them with an information letter describing the study background and interview procedure. Those who expressed interest in participating were contacted by telephone by a researcher (ETB, MK) to schedule the baseline interview.

### Procedure

Baseline interviews were conducted on the day the patient had an appointment at the simulator to plan radiation treatment or received their first radiation treatment. The follow-up interview took place on patients' last day of radiotherapy. To limit patient burden, the interviews were conducted either prior to or following simulator or first (baseline) and final treatment (follow-up), depending on patients' preferences. The interviews were conducted at the Department of Radiotherapy of the AMC by two researchers (ETB, MK) not involved in the patients' clinical care. Wherever possible, both interviews were conducted by the same interviewer (92% of all interviews) to enable consistency of the interview procedure at baseline and follow-up.

Items were derived from the 30-item EORTC QLQ-C30 [17], a HRQoL instrument widely used in European clinical trials [18]. To limit patient burden, we conducted a pilot study aimed at selecting items covering both global and specific content, including physical, psychological and social dimensions [16]. The following items resulted from this pilot study: 1) Do you have any trouble taking a short walk outside of the house?; 2) Have you had pain? 3) Were you tired?; 4) Did you worry? 5) Has your physical condition or medical treatment interfered with your social activities? 6) How would you rate your overall health during the past week? 7) How would you rate your overall quality of life during the past week?. In accordance with the EORTC QLQ-C30, a one-week time frame was employed. The first five items have four response categories: (1) not at all, (2) a little, (3) quite a bit, and (4) very much. The latter two items ask patients to rate their overall health and overall QoL on a 7-point Likert scale ranging from (1) very poor to (7) excellent.

To examine the cognitive processes that patients use in evaluating their QoL, we used the Three-Step Test Interview (TSTI) [19] combining cognitive think-aloud interviewing and verbal probing techniques [20]. As suggested in Willis' manual for cognitive interviewing [21], we started each interview with an exercise to acquaint participants with the think-aloud procedure. In this exercise, patients were asked to visualise their home and think out loud what they were seeing and thinking while counting all the windows. When patients immediately provided a response without thinking aloud (for example "8 windows"), the interviewer again explained the think-aloud procedure and repeated the exercise. All patients were able to perform this exercise, after which the actual think-aloud interview commenced. In these interviews, patients were asked to read out loud each QoL item and corresponding response categories, and to subsequently verbalise the thought processes used in providing their score. Immediately after the think-aloud response to each item, we probed the patients to elicit more information about their cognitive processes, using probes based on the cognitive process models of Tourangeau et al. [11] and Rapkin & Schwartz [10]. The probes were particularly directed to the cognitive processes that were not spontaneously mentioned by the patient (see Table 1). Additionally, we posed non-leading probes such as "Could you tell me more about that?" to further clarify patients' responses. All interviews were audio recorded and transcribed verbatim. Since this study was not intrusive and based solely on self-reports, the Medical Ethics Committee (MEC) of the AMC provided exemption from seeking formal approval, as is standard practice for such studies.

### Data analysis

Qualitative analysis of all interviews was independently carried out by the two interviewers (ETB, MK) and started directly after a patient had completed both interviews. To provide an open account of the cognitive processes that patients use in evaluating their QoL, analysis started with an initial reading of the interview and summarizing its salient content. We used our qualitative analysis scheme [16] based on the cognitive process models of Tourangeau et al. [11] and Rapkin & Schwartz [10] for the subsequent coding of patients' cognitive processes. Additional file 1 illustrates the use of this analysis scheme by providing an interview excerpt that is coded according to the five cognitive processes. Relevant text fragments were electronically coded using MAXqda software [22].

After both interviews of each patient had been coded independently by the two researchers (ETB, MK), they discussed their findings. In case of differences, agreement was achieved through negotiated consensus [23]. Once agreement was established about the assigned codes related to the underlying cognitive processes per item for both interviews of a single patient, the assumption of consistency in the content of the cognitive processes underlying QoL appraisal over time was examined. To that end, the researchers independently determined whether the content of each cognitive process was similar at baseline and follow-up, or rather changed over time. Since each response to each questionnaire item is unique, we were not able to draw up stringent guidelines in determining (dis)similarity over time in the content of each cognitive process. For the most part, (dis)similarity in the content per cognitive process was evident. The following example exemplifies similar content of the cognitive process comprehension/frame of reference of 'a short walk' (item 1): *"A short walk is walking to my office" *(Baseline); *"A short walk is going to work by foot." *(Follow-up) [Female, 37 years, breast cancer]. Conversely, an example of dissimilarity in the content of the cognitive process comprehension/frame of reference of 'a short walk' is: *"A short walk is walking for about half an hour." *(Baseline); *"[A short walk] is walking from the parking lot to the entrance of the hospital." *[100 metre; ETB] (Follow-up) [Female, 59 years, gynaecological cancer]. In cases where (dis)similarity appeared less evident, the two researchers reached a decision by discussing the likelihood of both similarity and dissimilarity in the content of the cognitive process concerned. Frequently, these discussions yielded a mutually agreed conclusion about (dis)similarity of the content of the cognitive process concerned. If doubt remained, we labelled the comparison of the content of a cognitive process over time as similar. We adopted this conservative approach to protect against a possible negative bias. Again, all findings were discussed and consensus negotiated in case of differences. Additional file 2 provides examples of similarity and dissimilarity in the content of all five cognitive processes for all seven items. All codes and subsequent analyses were discussed with FvZ and MS throughout the period of data collection and analysis.

To examine whether we could discern patterns of (dis)similarity across and within patients, we combined the assigned labels related to either similarity or dissimilarity in the content of each cognitive process over time with patient characteristics (i.e. gender, age, tumor site, and length of interval between patient's baseline and follow-up interview) in MAXqda software. Likewise, to examine possible patterns of (dis)similarity across items, these assigned labels were combined with each individual item.

## Results

### Participants

Ninety-two eligible patients were asked to participate. Thirty-one patients (34%) refused explaining they considered it too burdensome to be interviewed prior to and after radiation treatment. Sixty-one patients (66%) gave written informed consent, of whom 50 patients (54%) completed both interviews. Ten patients were unable to complete the follow-up interview due to severe health deterioration, and one patient could not be interviewed at follow-up due to logistical problems. The mean number of days between both interviews was 47 days (SD 11.7, range 27-82). Table 2 depicts the characteristics of the 50 patients who completed both the baseline and follow-up interview (median age 60 years, SD 11.2, range 35-85).

**Table 2:** Patient characteristics

	No of patients
Gender:	
Men	24
Women	26

Age (years):	
30-39	2
40-49	8
50-59	15
60-69	16
70-79	6
≥ 80	3

Tumor site:	
Bladder	4
Breast	10
Colorectal	4
Esophageal	9
Gynecological	7
Lung	6
Prostate	10

Length of interval between baseline and follow-up interview	
(median)	
< 50 days	22
≥ 50 days	28

This study was part of a more extensive investigation of the cognitive processes underlying QoL change evaluations, consisting of two consecutively conducted studies. The abovementioned inclusion criteria and data collection procedure were employed in both studies. The baseline and follow-up interviews were administered in both studies, extended with transition questions (study 1) and thentest questions (study 2) respectively. In qualitative research, the sample size is based on the criterion of data saturation [24], i.e. data collection can be stopped when the last three units of analysis do not yield new information. In a prior study, we had found that the content of the cognitive processes cancer patients use to arrive at an answer to our seven questionnaire items was not constant, but instead differed per questionnaire item within patients [16]. For example, patients compared themselves with other patients in answering one item, and referred to their own functioning prior to cancer diagnosis in responding to another item. Likewise, patients differed per item in the way they prioritized and combined positive and negative samples, the way they arrived at their answer, and so forth. Therefore, we used the response to each questionnaire item (constituting the five cognitive processes) as our unit of analysis, rather than the individual patient. The cognitive processes underlying QoL appraisal were saturated at an early stage of data collection. However, to include a heterogeneous sample, we purposively selected 26 and 24 cancer patients undergoing radiotherapy for study 1 and 2 respectively. The study's sample of 50 patients combines both subsamples, and thus exceeds the criterion for data saturation.

During the baseline and follow-up interviews, 43 patients completed all seven items, six patients provided interpretable data for six items, and one patient for five items. This yielded 342 responses per time point (a total of 684 responses) suitable for qualitative analysis [43 patients × 7 items + 6 patients × 6 items + 1 patients × 5 items], which were analyzed according to the five distinct cognitive processes of our analysis scheme. The assessment of (dis)similarity of the cognitive processes over time yielded 1710 evaluations (342 comparisons of responses over time × five cognitive processes).

### (Dis)similarity in the cognitive processes underlying QoL appraisal over time

#### 1) Comprehension/frame of reference

Twelve patients could not provide a definition of the target construct at either baseline and/or follow-up. For these items, we could not examine whether comprehension/frame of reference was similar or rather changed over time. Therefore, (dis)similarity in this cognitive component could be assessed for 330 out of 342 (96%) comparisons of responses over time. The content of the cognitive process comprehension/frame of reference changed between baseline and follow-up in 188 out of 330 comparisons of responses (57%) (Table 3). This change in the meaning patients attach to the target construct was primarily found in the items consisting of two target constructs, i.e. assessment of 'trouble' taking a 'short walk' (item 1; N = 35 out of 46 (76%) comparisons of responses) and 'interference' in 'social activities' (item 5; N = 34 out of 48 (71%) comparisons of responses). The following interview excerpts illustrate a change in the definition of a 'short walk', which is defined at baseline as a walk of "30 minutes, an hour", whereas at follow-up a short walk "is about 10 minutes". The patient's definition of 'trouble' remains similar over time, i.e. feeling tired:

**Table 3:** Dissimilarity per cognitive process underlying QoL appraisal over time for 7 EORTC QLQ-C30 items

	1 (trouble - short walk)	2 (pain)	3 (fatigue)	4 (worry)	5 (interference - social activities)	6 (overall health)	7 (overall QoL)	Total dissimilarity
**Comprehension/Frame of reference**	Number of responses: 46	Number of responses: 49	Number of responses: 48	Number of responses: 43	Number of responses: 48	Number of responses: 47	Number of responses: 49	Total number of responses: 330
								
	Trouble: 17	24	27	16	Interference: 16	27	25	188 (57%)
	Walk: 6				Social activities: 4			
	Both: 12				Both: 14			
								
	Dissimilarity in trouble and/or walk: 35				Dissimilarity in interference and/or social activities: 34			

**Retrieval/Sampling strategy**	Number of responses: 50	Number of responses: 49	Number of responses: 48	Number of responses: 49	Number of responses: 50	Number of responses: 47	Number of responses: 49	Total number of responses: 342
								
	Content: 39	Content: 45	Content: 47	Content: 41	Content: 45	Content: 45	Content: 49	Content: 311 (91%)
	Concept: 35	Concept: 27	Concept: 39	Concept: 26	Concept: 36	Concept: 39	Concept: 44	Concept: 246 (72%)

**Standards of comparison**	Number of responses: 50	Number of responses: 49	Number of responses: 48	Number of responses: 49	Number of responses: 50	Number of responses: 47	Number of responses: 49	Total number of responses: 342
								
	11	27	29	25	14	25	21	152 (44%)

**Judgment/Combinatory algorithm**	Number of responses: 20	Number of responses: 36	Number of responses: 28	Number of responses: 37	Number of responses: 28	Number of responses: 37	Number of responses: 34	Total number of responses: 220
								
	4	18	12	22	16	26	15	113 (51%)

**Reporting and response selection**	Number of responses: 50	Number of responses: 49	Number of responses: 48	Number of responses: 49	Number of responses: 50	Number of responses: 47	Number of responses: 49	Total number of responses: 342
								
	10	16	19	25	20	25	26	141 (41%)

##### Example 1

###### Do you have any trouble taking a short walk outside of the house?

**Baseline answer: not at all**; *"I do not experience any limitations, I just continue my normal life. To be honest, I do not walk that often. And if I go for a walk, I go somewhere by car to take a walk with a friend for pleasure. (...) I would say a short walk is about 30 minutes, an hour. And I do not have trouble with that, because it does not tire me."*

**Follow-up: not at all**; *"No, not really. A short walk is about 10 minutes and I manage walking that long without trouble. But if I have to walk a distance that is beyond a 10 minute walk, I get tired immediately. But a short walk is not troublesome."*

[Female, 49 years, breast cancer]

Conversely, the following excerpts are an example of change in the definition of 'trouble'; at baseline the patient defines trouble as a physical limitation, whereas at follow-up trouble is defined as a mental state. The meaning she attaches to the construct 'short walk' remains similar between the baseline and follow-up assessment, i.e. going out to do groceries:

##### Example 2

###### Do you have any trouble taking a short walk outside of the house?

**Baseline answer: not at all**; *"I go for a walk everyday. A short walk is to take a walk up and down the stores to get bread and some other groceries. (...) I do not have trouble with this everyday walking, but taking a walk in the dunes would be troublesome to me. Trouble is having to walk from the top downwards, because of my knee injury."*

**Follow-up: a little**; *"I go out shopping everyday, just to get some groceries. I walk to the drugstore for example, and back home again. (...) I do not have trouble going out and taking a walk physically, but there were days I had limbs like lead. I really did not want to go out on the street during those days, mentally."*

[Female, 61 years, bladder cancer]

#### 2) Retrieval/sampling strategy

In all 684 responses, we could distinguish patients' sampled experiences. (Dis)similarity in retrieval/sampling strategy could thus be assessed for all 342 comparisons of responses over time. Patients retrieved different information in 311 out of 342 comparisons of responses (91%) (Table 3). At follow-up, the majority of patients retrieved experiences from their radiotherapeutic treatment (N = 208), whereas, self-evidently, they did not at baseline. For example:

##### Example 3

###### How would you rate your overall health during the past week?

(range 1 (very poor) - 7 (excellent)

**Baseline answer: 4**; *"I have more trouble taking a walk, and I experience a bit more difficulty doing household chores. I am no longer able to clean the windows or sponge down the doors for my wife. But I am still able to vacuum the house. I definitely got a lot older."*

**Follow-up answer: 5**; *"My health deteriorated during the radiotherapeutic treatment. I feel more tired, I have a burning feeling inside, and my medication intake has increased. And I feel somewhat constrained, I have to undergo the radiation treatment every day. It's not that I can skip a treatment."*

[Male, 79 years, lung cancer]

Originally, we defined dissimilarity in retrieval/sampling strategy in the strictest sense, i.e. when the content of the samples differed over time. However, since it is unlikely that patients retrieve the exact same experiences during the baseline and follow-up assessment, we re-assessed dissimilarity in this cognitive component by not focusing on change in the content of the samples used, but rather on the concept the samples stem from. Consequently, the sampling strategy in the interview excerpts cited below was labelled similar over time, since the samples were derived from the same concept, i.e. pain as a result of cancer treatment. Based on concept instead of content of the samples used, patients' sampling strategy changed in 246 out of 342 comparisons of responses (72%) (Table 3).

##### Example 4

###### Have you had pain?

**Baseline answer: a little**; *"When I think of the breast surgery, I did experience some pain. My breast was tender, and the wound became inflamed which made my breast even more sore."*

**Follow-up answer: a little**; *"I did feel the radiation. It prickled and caused stings in my breast. And the skin underneath my breast is open, which is very unpleasant because I prefer to wear a bra."*

[Female, 48 years, breast cancer]

#### 3) Standards of comparison

The reference groups patients used to judge their functioning could be discerned in all 684 responses, yielding 342 comparisons of responses over time. The reference group used changed in 152 out of 342 comparisons of responses (44%) (Table 3). In the majority of responses at baseline, patients verbalized a comparison to their own functioning prior to cancer diagnosis and treatment (N = 242). In 168 responses at follow-up, patients used the same reference group, whereas in the remaining responses they used a different one, including their functioning during the first weeks of radiotherapy (N = 33), other cancer patients (N = 11), expectations about future functioning (N = 10) or other (N = 43), for example people the same age.

The following excerpts exemplify a patient who assessed his level of fatigue at baseline using his own functioning prior to cancer diagnosis and treatment as standard of comparison, whereas at follow-up he compared his current fatigue with his level of fatigue during the first weeks of radiotherapy:

##### Example 5

###### Were you tired?

**Baseline answer: not at all**; *"I was not tired at all. I am working out how I am feeling now, and I contrast this to the times I can remember I was feeling tired. When I feel tired, I always have weary legs. And that happens when I have worked long and hard, after physical strain. And that isn't the case now, thus no, I do not feel tired at all."*

**Follow-up answer: quite a bit**; *"I was not very tired during the radiotherapeutic treatment. When compared with the first weeks of treatment, I only began to experience fatigue in the last two weeks. At night I lay awake, which makes you feel tired during the day. In the beginning of treatment, I did not have trouble sleeping."*

[Male, 59 years, prostatic cancer]

Again, the patient in the following example expresses a comparison to her QoL prior to cancer diagnosis and treatment at baseline, whereas at follow-up she uses other (cancer) patients as comparator:

##### Example 6

###### How would you rate your overall quality of life during the past week?

(range 1 (very poor) - 7 (excellent)

**Baseline answer: 6; ***"I have something inside of me that does not belong there. Tomorrow they will make a scan to see whether I have metastases. And I have to admit, that is pretty scary. (...) [My quality of life] is a 6. If I were not in this situation, I would choose a 7."*

**Follow-up answer: 7'; "***I have to say a 7. True, I have cancer and I suffer discomfort. But if I compare myself to the other patients I see here in the hospital, I really cannot complain."*

[Female, 47 years, colorectal cancer]

#### 4) Judgment/combinatory algorithm

This cognitive process is of relevance when patients retrieve both positive samples (e.g. *"During the day I don't suffer from pain in my oesophagus."*) and negative samples (e.g. *"My oesophagus feels like a raw wound when I eat something." *[Male, 49 years, oesophageal cancer]. In the subsequent prioritization and combination of positive and negative samples, patients can emphasize either the positive or negative experiences, or find a balance between both in arriving at an answer. Patients retrieved positive and negative samples in 220 responses at both baseline and follow-up, resulting in 220 comparisons of responses over time. The prioritization and combination of retrieved samples changed in 113 out of 220 comparisons of responses over time (51%) (Table 3). In the majority of the responses at baseline, patients balanced between positive and negative samples (N = 91) or emphasized the positive samples (N = 87). At follow-up, part of the balanced combinatory algorithms shifted to an emphasis on the positive samples (N = 20) or negative samples (N = 19). The emphasis on the positive samples shifted, in part, to a balanced combinatory algorithm (N = 32) or to an emphasis on the negative samples (N = 31). The patient in the following example expresses worry about her cancer during both interviews, but at the same time emphasizes her positive outlook. At baseline, her positive outlook determines her answer, whereas at follow-up her worries outweigh her optimistic view.

##### Example 7

###### Did you worry?

**Baseline answer: a little**; *"I worry a little, considerable. But from the moment I heard I have cancer, I have the feeling everything will work out for the best."*

**Follow-up answer: very much**; *"Yes, I worry. I hope the radiation treatment has been successful. But I am very positive, I also told you that before the treatment started. I have a positive feeling about it, and I hope I will be able to keep that feeling."*

[Female, 46 years, gynaecological cancer]

In the above-mentioned example, the patient based her answer on the same samples but used a different judgment/combinatory algorithm. The following excerpts exemplify assessments of overall health in which the patient retrieves different samples at baseline and follow-up, albeit both positive and negative ones. However, at baseline the positive sample outweighs the negative one since the patient rates her health a '7' (i.e. excellent), whereas at follow-up the patient balances the positive and negative samples in rating her health a '6', which is "right in between" feeling a '5' after chemotherapy (negative sample) and feeling a '7' "in the last week before the next chemo" (positive sample):

##### Example 8

###### How would you rate your overall health during the past week?

(range 1 (very poor) - 7 (excellent)

**Baseline answer: 7**; *"That is an easy one, I go for a '7' [excellent]. I have had a very good week. (...) For me, health is being able to do everything you like. I do have several complaints you know, I have a knee injury, I have had foot surgery...But last week, we were out for dinner, and we enjoyed a lovely meal. So last week is definitely a '7'. "*

**Follow-up answer: 6**; *"My health is unstable. When I come home from chemotherapy I feel like a '5', I feel nauseated right after the treatment. But other than that, I do not feel sick. So I can not say my health is a '5'. On the other hand, it isn't a '7' either, because I only feel like a 7 in the last week before the next chemo. So I will opt for a '6', right in between."*

[Female, 61 years, lung cancer]

#### 5) Reporting and response selection

In all 684 responses, the patients explained how they arrived at their answer and chose the selected response category, yielding 342 comparisons of responses over time. The content of this cognitive process changed in 141 out of 342 comparisons of responses (41%) (Table 3). The way patients arrived at their answer is highly diverse, but often included patients' use of editing processes aimed at mitigating the initial response at either baseline or follow-up (N = 55). The patient in the following example uses such an editing process at follow-up, whereas at baseline he does not downplay the extent to which he worries:

##### Example 9

###### Did you worry?

**Baseline answer: quite a bit**; *"I never worry. My only worry now is, will they treat my illness in the best way possible? That is my worry, that they do not make a mistake. So, during the past week, I worried quite a bit."*

**Follow-up answer: not at all**; *"I do not worry about my wife and my children, my only worry is whether I still have cancer. Did the radiation treatment cure me? (...) I am overruling my worries, I have to believe that I am cured. So, I do not worry at all, but that is because I push my worries aside."*

[Male, 60 years, prostatic cancer]

In the following excerpts, the patient chose his answer without apparent cognitive consideration at baseline, whereas at follow-up the process of arriving at an answer involves deliberate reasoning:

##### Example 10

###### Have you had pain?

**Baseline answer: a little**; *"Let's say 'a little', since that's the first response option I see (...) I could have chosen 'quite a bit' just as well."*

**Follow-up answer: quite a bit**; *"I consider pain due to visiting a dentist as 'very much' pain. Since my current pain isn't as bad as toothache, I opt for 'quite a bit'."*

[Male, 78 years, prostatic cancer]

The questionnaire format allows patients to assess their overall health and QoL on a scale ranging from 1 (very poor) to 7 (excellent). Some patients interpreted this scale at one of the interviews as an incomplete evaluation scale ranging from 1-10. For example:

##### Example 11

###### How would you rate your overall health during the past week?

(range 1 (very poor) - 7 (excellent)

**Baseline answer: 7**; *"Well, 'excellent' is pushing things too far, but I dare to go for an '8'. But I see I cannot choose an '8' here, so then I will opt for a '7'. (...) I wish I could rate my health with an '8', because that's a little closer to '10'."*

**Follow-up answer: 4**; *"My health is pretty moderate at the moment, so I choose a '4' [response option right in the middle of the response scale]. (...) I am in pain, I am having a cold, and my nose is bleeding frequently. My health is decreased as a result of treatment, but they did warn me for that."*

[Male, 67 years, oesophageal cancer]

### Patterns of (dis)similarity in the cognitive processes underlying QoL appraisal

In the majority of responses the content of the QoL appraisal process changed over time, i.e. in 322 comparisons of responses (94%) the content of at least one cognitive process changed. Additionally, in each patient, the content of all five cognitive processes changed over time for a different number of the seven items, and was similar for the remaining items. However, dissimilarity in the content of each of the cognitive processes differed across and within patients, and was found to be unrelated to patient characteristics. Additionally, dissimilarity was unrelated to the questionnaire item, and dissimilarity of the preceding item, i.e. differences found in the content of a cognitive process for one item was not found to influence dissimilarity in the content of that same cognitive process for the subsequent item.

In contrast to changes in the QoL appraisal process over time, 20 comparisons of responses (6%) were based on similarity in the content of the QoL appraisal process over time, i.e. the content of all five cognitive processes remained constant from baseline to follow-up. These 20 comparisons of responses were generated by a heterogeneous group of 15 different patients. All seven items were answered at least once based on similar QoL appraisal processes over time. As with dissimilarity, similarity in the content of the QoL appraisal process over time was found to be unrelated to item and patient characteristics.

## Discussion

The interviews yielded 342 comparisons of baseline and follow-up responses, which were analyzed according to the five cognitive processes underlying QoL appraisal. The content of comprehension/frame of reference changed in 188 comparisons; retrieval/sampling strategy in 246; standards of comparison in 152; judgment/combinatory algorithm in 113; and reporting and response selection in 141 comparisons. Overall, in 322 comparisons of responses (94%) the content of at least one cognitive component changed over time. We could not discern patterns of (dis)similarity across and within patients and/or items. Thus, the assumption of consistency in the content of the cognitive processes underlying QoL appraisal over time was not found to be in line with the cognitive processes described by the respondents.

The limitations of this study should be noted. First, thirty-one patients refused participation, and severe health deterioration prevented ten patients to complete the follow-up interview. This might indicate that the most severely ill patients were not included. We cannot exclude the possibility that these patients might have described different cognitive processes. However, to ensure a heterogeneous sample and wide variation in cognitive processes used in evaluating QoL, we sampled the patients purposively based on gender, age, tumor site and length of radiation treatment. Additionally, our questionnaire items are derived from the cancer-specific EORTC QLQ-C30, aimed to assess cancer patients' functioning and wellbeing independent of their treatment. Therefore, one could expect similar results for cancer patients undergoing cancer treatment other than radiotherapy, e.g. chemotherapy or surgery. Second, the extent to which think-aloud interviews truly reflect patients' cognitive processes can be questioned. Therefore, we not only asked patients to think aloud while answering the QoL items, but additionally probed them for clarification or during pauses in which they did not think aloud to capture patients' cognitive processes as comprehensively as possible. In addition, we probed the patients concurrently after their think-aloud response to each item instead of retrospectively after administering all questionnaire items to diminish the chance of participants reconstructing their answering process instead of recalling it [16]. When probing concurrently, the cognitive processes that patients use might be influenced by the probing of the preceding item. However, since we could not detect a pattern in the content of the cognitive processes used, such order effects are likely negligible. Third, for this study we have selected seven QoL items that best reflect the multidimensional character of QoL, i.e. physical, psychological, and social functioning. However, this heterogeneity might have induced differences in the content of the cognitive processes used. Future research should examine how (dis)similar the content of the cognitive processes over time is using questionnaire items addressing one specific domain.

We operationalized participants' cognitive processes according to the distinct cognitive components of the combined models of Tourangeau et al. [11] and Rapkin & Schwartz [10]. Our results might thus be perceived as a product of these models. However, in our prior study in which we have combined both models in developing a qualitative analysis scheme, we started the analysis with an initial reading of the interview and summary of its salient content to provide an open account of the cognitive processes used. Additionally, we actively searched for information that would not fit in or run counter to these models. The results indicated that the combined models comprehensively capture the cognitive processes underlying QoL appraisal [16].

According to Rapkin & Schwartz' QoL appraisal model, patients are assumed to answer all individual questionnaire items at one time point using the same cognitive processes, e.g. respondents use the same reference group(s) in answering all items of an entire questionnaire. At an earlier stage we found that the content of the cognitive processes differed per item within patients [16]. This study shows that the same holds for differences in the content of the cognitive processes used over time, i.e. the content of each of the cognitive processes over time differed within the same patient across items. For example, a patient may use the same standards of comparison over time in answering three items, and may use a variety of different standards of comparison in the other four. Moreover, the content of a cognitive process underlying a particular item was not found to influence the content of that cognitive process for the subsequent item. This finding is in line with a quantitative study conducted by Fayers et al. [25] according to which changes in reference groups used at successive assessments of overall QoL appeared randomly.

Rapkin & Schwartz [10] mapped change in the content of each of the cognitive processes constituting their QoL appraisal model to one of the specific types of response shift [26], i.e. change in frame of reference is related to reconceptualization (a redefinition of the target construct), change in sampling strategy and combinatory algorithm to reprioritization (a change in individual's values), and change in standards of comparison to recalibration (a change in individual's internal standards). Although a distinction is made between these different types of response shift, they are likely to be interdependent and to co-occur [27]. Our results support this interconnection, since in the majority of the comparisons of responses, we found changes in the content of multiple cognitive processes underlying one item, for example both reconceptualization and reprioritization.

The extent to which change in the content of the underlying cognitive processes resulted in invalid QoL comparisons over time was found to vary and could not be established unequivocally. For example, in the abovementioned Example 1, change in the patient's definition of a short walk, clearly renders a comparison over time incompatible. Conversely, change in the standards of comparison used in Example 5 does not result in an apparently invalid comparison over time. At follow-up the respondent describes that his fatigue has increased during the last two weeks of radiotherapy. When comparing his assessment of fatigue at baseline ('not at all') and follow-up ('quite a bit'), the conclusion is warranted that this patient's level of fatigue has increased. QoL appraisal inherently involves subjective assessment and reflects the patient's perspective of his/her functioning at a given point in time. Change in the content of the cognitive processes underlying QoL appraisal over time may likely result from patients' adaptation to their changing health status, apart from chance fluctuations. Thus, from the patients' perspective, the interpretation of QoL scores over time is a reflection of 'true' change over time. However, in interpreting QoL scores over time in the context of clinical research, one needs to be aware of the fact that patients provide QoL assessments based on personally meaningful content of the underlying cognitive processes, that may not be consistent over time as the baseline and follow-up design assumes. An interesting way to move this line of research a step further is to confront patients with their answers to disentangle response shift from other adaptive mechanisms and random fluctuations [10].

Importantly, this study demonstrates change in the content of the QoL appraisal process over time at the *individual *level. However, numerous clinical studies, such as randomized clinical trials, have provided meaningful outcomes in the expected direction when measuring change in QoL at the *group *level [28,29]. Apparently, at the group level, dissimilarity in the content of an individual's QoL appraisal process does not seem to invalidate change outcomes. Our findings thus raise the question about how strict the assumption of consistency in the content of the QoL appraisal process over time needs to be adhered to at the group level.

Nonetheless, these qualitative findings show how the validity of prospective QoL assessment can be increased by enhancing a consistent interpretation of the item over time. Answering QoL questionnaire items can be a complex cognitive task for respondents, since this requires them to pass through each of the underlying cognitive processes [30,31]. A number of factors such as the wording of items and instructions accompanying a questionnaire, influence respondents' ability to accurately understand the item and to report the requisite information [32]. To stimulate unambiguous interpretation of items, instructions accompanying a questionnaire should invoke specific content of cognitive processes e.g. a particular frame of reference and reference group [25]. To illustrate, patients may be asked to think of their functioning as a result of radiation treatment in comparison to their own functioning prior to cancer diagnosis and treatment. Additionally, to diminish differences in the content of comprehension/frame of reference used, it is of importance to define the target construct as concretely as possible, for example assessing whether patients are experiencing trouble in taking a walk of one kilometre instead of a short walk. Moreover, differences in the content of the cognitive process comprehension/frame of reference was primarily found in the items consisting of two target constructs, i.e., 'trouble' taking a 'short walk' (item 1) and 'interference' in 'social activities' (item 5). Clearly, with two target constructs, the chance of changes in the content of comprehension/frame of reference doubles. Adapting these items such that they include only one target construct is likely to enhance similarity in the content of this cognitive process over time.

There is general agreement about the importance of obtaining patients' perspectives in understanding the impact of illness and its treatment. Studies including patient-reported outcomes such as QoL have yielded important findings relevant for researchers, clinicians and patients [33]. The usefulness of QoL measurement is thus beyond doubt. Our findings contribute to a better understanding of patient-reported QoL outcomes in building on theoretical frameworks describing the cognitive processes underlying QoL appraisal and the response shift literature, and underscore the previously documented recommendations to improve QoL questionnaire items to yield unambiguous responding.

## Conclusions

This is the first known study to qualitatively examine the assumption of consistency in the content of the distinct cognitive processes underlying QoL appraisal over time. The content of each of the five cognitive processes underlying QoL appraisal (i.e. comprehension/frame of reference, retrieval/sampling strategy, standards of comparison, judgment/combinatory algorithm, and reporting and response selection) was found to change over time. Overall, in 322 (94%) out of the 342 comparisons of responses over time, the content of at least one cognitive process changed. Additionally, we could not discern patterns of (dis)similarity since the content of each of the cognitive processes differed across and within patients and/or items. Thus, the assumption of consistency in the content of the cognitive processes underlying QoL appraisal over time was not found to be in line with the cognitive processes described by the respondents. In building on cognitive process models and the response shift literature, this study contributes to a better understanding of patient-reported QoL appraisal over time.

## Competing interests

The authors declare that they have no competing interests.

## Authors' contributions

MAGS, MRMV, FJvZ, and CCEK designed the study and wrote the research proposal. EFTB and MAK conducted the interviews, and coded and analyzed all data. The codes and subsequent analyses were discussed with FJvZ and MAGS. EFTB wrote the first draft. All authors commented on and contributed to the final draft.

## Supplementary Material

Additional file 1 **Illustration of the cognitive processes constituting the qualitative analysis scheme**. Interview excerpt which is coded according to the five cognitive processes underlying QoL appraisal to illustrate the use of our analysis scheme based on the cognitive process models of Tourangeau et al. (2000) and Rapkin & Schwartz (2004). Click here for file

Additional file 2 **Examples of similarity and dissimilarity in the content of the five cognitive processes underlying the seven QoL items**. Illustration of similarity and dissimilarity in the content of the five cognitive processes between baseline and follow-up for all seven QoL items. Click here for file
