# Supplementary material for: A 'short walk' is longer before radiotherapy than afterwards: a qualitative study questioning the baseline and follow-up design
Source: Health Qual Life Outcomes. 2010 Jul 16;8:69. doi: 10.1186/1477-7525-8-69 (PMC2915972; doi:10.1186/1477-7525-8-69)
Supplement: Additional file 1 — Illustration of the cognitive processes constituting the qualitative analysis scheme. Interview excerpt which is coded according to the five cognitive processes underlying QoL appraisal to illustrate the use of our analysis scheme based on the cognitive process models of Tourangeau et al. (2000) and Rapkin & Schwartz (2004). [file 1477-7525-8-69-S1.DOC]

# Additional files

**Additional file** 1 - Illustration of the cognitive processes constituting the qualitative analysis scheme

| How would you rate your overall quality of life during the past week? | | | |
| --- | --- | --- | --- |
| (range 1 (very poor) – 7 (excellent) | | | |
| *A. "Quality of life comprises your health, and that you don't have problems financially.* | | | |
| *B. I also consider our children and grandchildren as important for my quality of life.* | | | |
| *C. (…) But I am not completely recovered from surgery yet.* | | | |
| *D. And as a result of that, my wife and I do not have a sexual relationship anymore.* | | | |
| *E. But my wife takes a glass of wine to bed now, and I bring a bottle of beer.* | | | |
| *F. We sit in bed together, very comfortable, and we talk or watch some television.* | | | |
| *G. So despite my impairments, my quality of life is good.* | | | |
| *H. Excellent is exaggerated, so I go for a 6.* | | | |
| [Male, 64 years, prostatic cancer] | | | |
|  |  |  |  |
| **Cognitive component** | **Code** | **Description code** | **Text fragment** |
| Comprehension / Frame of reference | Definition | Meaning of target construct - quality of life | Health (A.) |
|  |  |  | Not having problems financially (A.) |
|  |  |  | Children and grandchildren (B.) |
|  |  |  | Being completely recovered from surgery (C.) |
|  |  |  | Having a sexual relationship (D.) |
|  |  |  | Sitting in bed together, very comfortable (F.) |
| Retrieval / Sampling strategy | Positive sample | Retrieval of positive information | But my wife takes a glass of wine to bed now, and I bring a bottle of beer. We sit in bed together, very comfortable, and we talk or watch some television. (E.-F.) |
|  | Negative sample | Retrieval of negative information | But I am not completely recovered from surgery yet. And as a result of that, my wife and I do not have a sexual relationship anymore. (C.-D.) |
| Standards of comparison | Self - prior to cancer diagnosis and treatment | Comparison with own functioning prior to cancer diagnosis and treatment | But I am not completely recovered from surgery yet. And as a result of that, my wife and I do not have a sexual relationship anymore. (C.-D.) |
| Judgment / Combinatory algorithm | Emphasis - positive | Emphasis on positive sample(s) in prioritizing and combining positive and negative samples | So, despite the impairments, my quality of life is good (G.) |
| Reporting and response selection | Response selection | Explanation of choice for the selected response category | Excellent is exaggerated, so I go for a 6 (H.) |
