# Supplementary material for: A 'short walk' is longer before radiotherapy than afterwards: a qualitative study questioning the baseline and follow-up design
Source: Health Qual Life Outcomes. 2010 Jul 16;8:69. doi: 10.1186/1477-7525-8-69 (PMC2915972; doi:10.1186/1477-7525-8-69)
Supplement: Additional file 2 — Examples of similarity and dissimilarity in the content of the five cognitive processes underlying the seven QoL items. Illustration of similarity and dissimilarity in the content of the five cognitive processes between baseline and follow-up for all seven QoL items. [file 1477-7525-8-69-S2.DOC]

# Additional files

### Additional file 2 – Examples of similarity and dissimilarity in the content of the five cognitive processes underlying the seven QoL items1

| **Comprehension / Frame of reference**  **- (dis)similarity in the definition a patient attaches to the target construct -** | **Baseline** | **Follow-up** | **(Dis)similarity** |
| --- | --- | --- | --- |
| **Item 1 - Do you have any trouble taking a short walk outside of the house?** | "A short walk is walking to my office." (Not at all) | "A short walk is going to work by foot." (Not at all) | Similarity [Female, 37 years, breast cancer] |
|  | "A short walk is walking for about half an hour." (Not at all) | "[A short walk] is walking from the parking lot to the entrance of the hospital." (A little) | Dissimilarity [Female, 59 years, gynaecological cancer] |
| [Range: Not at all - A little - Quite a bit - Very much] | "Trouble taking a walk is due to my hip injury." (Not at all) | "I have trouble taking a walk because of a hip injury." (Not at all) | Similarity [Female, 61 years, lung cancer] |
|  | "Trouble taking a short walk is due to a lack of time." (Not at all) | "Physically, I do not have any trouble taking a walk." (Not at all) | Dissimilarity [Male, 66 years, prostatic cancer] |
| **Item 2 - Have you had pain?** | "I have almost continuous pain in my oesophagus when I eat and swallow." (A little) | "I have a burning pain in my oesophagus, especially when I eat and drink. It is almost continuous now." (Quite a bit) | Similarity [Male, 67 years, oesophageal cancer] |
| [Range: Not at all - A little - Quite a bit - Very much] | "I didn't need pain medication after surgery, so I wasn't in pain." (Not at all) | "I wasn't in mental pain." (Not at all) | Dissimilarity [Female, 49 years, breast cancer] |
| **Item 3 - Were you tired?** | "Tired is when I come home from a day at work." (A little) | "Feeling tired is like I come home from work." (Quite a bit) | Similarity [Male, 57 years, prostatic cancer] |
| [Range: Not at all - A little - Quite a bit - Very much] | "I am taking up with it [cancer diagnosis] in my mind, which decreases my energy." (A little) | "I have done too much and I have rested too little." (A little) | Dissimilarity [Female, 49 years, breast cancer] |
| **Item 4 - Did you worry?** | "Being down casted." (A little) | "I am not down casted." (Very much) | Similarity [Female, 46 years, gynaecological cancer] |
| [Range: Not at all - A little - Quite a bit - Very much] | "Worrying is to feel completely worn out." (Quite a bit) | "[Worrying is] feeling scared." (Quite a bit) | Dissimilarity [Female, 57 years, oesophageal cancer] |
| **Item 5 - Has your physical condition or medical treatment interfered with your social activities?** | "My social activities are the hobbies I do together with a group of people, such as Nordic-walking." (A little) | "Nordic-walking with a group, that is one of my social activities." (Not at all) | Similarity [Female, 51 years, breast cancer] |
| [Range: Not at all - A little - Quite a bit - Very much] | "I am a very busy person. My social activities revolve around my work, and getting people to work for me." (Not at all) | "My social activities concern my relationship with my wife, and our sexual relationship." (Very much) | Dissimilarity [Male, 60 years, prostatic cancer] |
|  | "[Interference is] that I am no longer able to do everything alone, I need help from my wife." (Quite a bit) | "Interference is that I am no longer independent." (Very much) | Similarity [Male, 64 years, bladder cancer] |
|  | "I feel interfered because I get so tired of telling the same story about my cancer over and over again to different people." (Not at all) | "[Interference is] feeling sick." (Not at all) | Dissimilarity [Female, 61 years, lung cancer] |
| **Item 6 - How would you rate your overall health during the past week?** | "Health is feeling well, that you don't feel sick." (5) | "[Health is] feeling well. I don't feel sick." (6) | Similarity [Female, 59 years, breast cancer] |
| [Range: 1 (very poor) - 7 (excellent)] | "Health is being independent, and having no pain." (6) | "I am healthy mentally, and I have a positive outlook." (5) | Dissimilarity [Female, 66 years, gynaecological cancer] |
| **Item 7 - How would you rate your overall quality of life during the past week?** | "Quality of life is being able to do everything what I want to do, and not being restricted by anything." (5) | "Quality of life is being able to do what I want to do without any limitations." (3) | Similarity [Female, 51 years, colorectal cancer] |
| [Range: 1 (very poor) - 7 (excellent)] | "Quality of life is happiness. Having a normal day, being able to eat for example. Just enjoying life." (7) | "Quality of life is the way everyone here in the hospital is supporting me. Everyone is friendly, and nothing is too much trouble for them." (7) | Dissimilarity [Male, 52 years, oesophageal cancer] |
| **Retrieval / Sampling strategy**  **- (dis)similarity in the concept patient’s retrieved samples stem from -** | **Baseline** | **Follow-up** | **(Dis)similarity** |
| **Item 1 - Do you have any trouble taking a short walk outside of the house?** | "I don't have trouble taking a walk. You see, I live nearby the market, so automatically I take a walk there every day." (Not at all) | "I stroll the market every day, so I haven't got trouble taking a walk." (Not at all) | Similarity [Female, 54 years, breast cancer] |
| [Range: Not at all - A little - Quite a bit - Very much] | "I have trouble taking a walk because I get pain in the back very easily." (A little) | "I just don't feel like taking a walk these days. I have to go out every day now because of the radiation treatment, so if I prefer staying at home now." (A little) | Dissimilarity [Male, 68 years, oesophageal cancer] |
| **Item 2 - Have you had pain?** | "After surgery, it hurts to go to the bathroom." (A little) | "Going to the bathroom caused more and more pain in the course of radiation treatment." (Quite a bit) | Similarity [Male, 78 years, prostatic cancer] |
| [Range: Not at all - A little - Quite a bit - Very much] | "My hip and knee no longer function as in the old days." (A little) | "When I eat something, I get a burning feeling in my oesophagus." (A little) | Dissimilarity [Male, 79 years, lung cancer] |
| **Item 3 - Were you tired?** | "Before this cancer diagnosis, I have had a stroke. Since that stroke, I have a lack of energy." (Very much) | "Since my stroke, I am so very tired. The radiation treatment only increased this lack of energy." (Very much) | Similarity [Male, 58 years, prostatic cancer] |
| [Range: Not at all - A little - Quite a bit - Very much] | "I was invited for a party last weekend, so I went to bed in the middle of the night." (Very much) | "I was tired after the chemotherapeutic treatments, I did not have any energy left." (Quite a bit) | Dissimilarity [Male, 49 years, oesophageal cancer] |
| **Item 4 - Did you worry?** | "I worry about the surgery I have to undergo after my radiation treatment. But I completely trust my doctor that everything will turn out fine." (A little) | "I dread the surgery, but I am in good hands here. I have every confidence in my doctor." (Not at all) | Similarity [Male, 75 years, oesophageal cancer] |
| [Range: Not at all - A little - Quite a bit - Very much] | "I worry about the actual radiation treatment, how things are at that department." (Quite a bit) | "I worry about a possible recurrence of cancer." (A little) | Dissimilarity [Female, 71 years, gynaecological cancer] |
| **Item 5 - Has your physical condition or medical treatment interfered with your social activities?** | "Sports and taking a walk are part of my social activities. It's not that I completely quit doing it, but I do it less frequently." (Quite a bit) | "At the moment I am no longer able to do the social activities I used to do, such as sporting." (Very much) | Similarity [Male, 56 years, lung cancer] |
| [Range: Not at all - A little - Quite a bit - Very much] | "When I was younger, I used to make house-to-house collections. But I am no longer able to do so." (Not at all) | "I feel limited by the noise my neighbour [in the nursing home] produces at night." (Quite a bit) | Dissimilarity [Female, 85 years, gynaecological] |
| **Item 6 - How would you rate your overall health during the past week?** | "Naturally, I don't like having cancer, but I don't feel sick." (5) | "I know I have cancer, but I don't feel sick." (6) | Similarity [Female, 59 years, breast cancer] |
| [Range: 1 (very poor) - 7 (excellent)] | "I strained my back two years ago, and I think that has caused some damage to one of my nerves." (6) | "I was tired last Saturday and Sunday." (6) | Dissimilarity [Male, 66 years, prostatic cancer] |
| **Item 7 - How would you rate your overall quality of life during the past week?** | "Since surgery, the wound is causing me pain." (6) | "I have had a rough week due to radiotherapy, I really felt miserable." (3) | Similarity [Male, 57 years, oesophageal cancer] |
| [Range: 1 (very poor) - 7 (excellent)] | "My children are doing fine, and I am happily married for 41 years." (7) | "All too often I have to go out of bed at night to go to the bathroom." (6) | Dissimilarity [Female, 61 years, bladder cancer] |
| **Standards of comparison**  **- (dis)similarity in reference group against which a patient judges his/her functioning -** | **Baseline** | **Follow-up** | **(Dis)similarity** |
| **Item 1 - Do you have any trouble taking a short walk outside of the house?** | "I already suffered from neuropathy, but the pain has increased as a result of treatment, which makes walking troublesome." (Quite a bit) | "The neuropathy in my right leg and foot has got worse due to the hormonal therapy." (A little) | Similarity [Male, 58 years, prostatic cancer] |
| [Range: Not at all - A little - Quite a bit - Very much] | "I haven't got trouble taking a walk. I don't feel tired and I am doing fine." (Not at all) | "In the course of radiotherapy, the fatigue increased. I started to get really tired during these last two weeks." (Not at all) | Dissimilarity [Female. 46 years, gynaecological cancer] |
| **Item 2 - Have you had pain?** | "They removed some lymph nodes at the right side, and that caused a little pain now and then." (Not at all) | "I have pain going to the bathroom now, but that's due to radiotherapy of course." (Quite a bit) | Similarity [Male, 64 years, prostatic cancer] |
| [Range: Not at all - A little - Quite a bit - Very much] | "I feel miserable, but there are people who are in more pain than I am." (Not at all) | "At the moment I am not in pain, but I have had appendicitis when I was young." (A little) | Dissimilarity [Female, 85 years, gynaecological cancer] |
| **Item 3 - Were you tired?** | "I am more tired now than prior to the start of cancer treatment." (A little) | "I am tired quite frequently. I didn't feel that way before the start if treatment." (A little) | Similarity [Female, 67 years, oesophageal cancer] |
| [Range: Not at all - A little - Quite a bit - Very much] | "I live under a bit of stress lately, since I have to take care of my mother who has become ill." (A little) | "I am extremely tired at the moment. When I compare this to the start of radiotherapy, my fatigue increased gradually." (Very much) | Dissimilarity [Female, 59 years, gynaecological cancer] |
| **Item 4 - Did you worry?** | "I am a heartily person, and I worry whether I will still be able to eat and drink those things that I am used to after surgery." (Quite a bit) | "I worry about what will remain of my eating and drinking habits after surgery." (Quite a bit) | Similarity [Male, 67 years, oesophageal cancer] |
| [Range: Not at all - A little - Quite a bit - Very much] | "I have changed since the cancer diagnosis, I was always in the best of spirits. But at this moment, I worry quite a bit." (Quite a bit) | "I know my chances of survival are big. But I keep thinking, other people I knew were having these same chances, but they did die anyway." (Very much) | Dissimilarity [Male, 57 years, prostatic cancer] |
| **Item 5 - Has your physical condition or medical treatment interfered with your social activities?** | "The only limitation is that I used to jog, but now I quit doing that." (A little) | "There are a couple of things which I used to do, but haven't done now, such as going to work or going to a birthday party." (6) | Similarity [Female, 50 years, breast cancer] |
| [Range: Not at all - A little - Quite a bit - Very much] | "I was able to keep carrying out my normal social activities." (Not at all) | "Compared to one of my friends, who is ill too, I am totally fine. I was able to pay him a visit, whereas he is as good as dead." (Not at all) | Dissimilarity [Male, 58 years, prostatic cancer] |
| **Item 6 - How would you rate your overall health during the past week?** | "Normally, if I were healthy, I would have chosen a '7'. But at the moment [my health] isn't excellent of course." (6) | "Naturally, [my health] isn't excellent at the moment, so I opt for a '5'. (5) | Similarity [Male, 64 years, colorectal cancer] |
| [Range: 1 (very poor) - 7 (excellent)] | "I know there are people my age, healthy people, whose capability is less than mine. That is normative for me, because I am able to do more than they do." (5) | "My health is decreased compared to how it used to be." (5) | Dissimilarity [Male, 64 years, prostatic cancer] |
| **Item 7 - How would you rate your overall quality of life during the past week?** | "Compared to my normal life, this situation is not something I would opt for." (5) | "Even laughing makes me tired now, and I feel that's absurd. This is not the way I usually am, and I don't want to be like this." (2) | Similarity [Female, 35 years, gynaecological cancer] |
| [Range: 1 (very poor) - 7 (excellent)] | "The cancer diagnosis made me realize that I actually am a lucky dog. I have so much that other people don't have: I have healthy children and a caring husband." (6) | "I cannot give a '7', because I feel tired now due to the radiation treatment and I don't like that my skin is irritated now." (6) | Dissimilarity [Female, 48 years, breast cancer] |
| **Judgment / Combinatory algorithm**  **- (dis)similarity in the way a patient prioritizes and combines the retrieved samples -** | **Baseline** | **Follow-up** | **(Dis)similarity** |
| **Item 1 - Do you have any trouble taking a short walk outside of the house?** | "[Taking a walk] can be tiring, but that's not always the case. It depends on the activities I carried out previous to it." (A little; balance) | "I don't have trouble taking a short walk, but if the walking distances are long I have to return by car." (A little; balance) | Similarity [Female, 47 years, colorectal cancer] |
| [Range: Not at all - A little - Quite a bit - Very much] | "Since I need to go to the bathroom this often, I can't stay outside for a long time. But I do go for a walk everyday." (Not at all; positive) | "I have to go to the bathroom in almost every shop I pass, but other than that, I don't have trouble." (A little; balance) | Dissimilarity [Female, 61 years, bladder cancer] |
| **Item 2 - Have you had pain?** | "I have pain in my left upper arm, but I don't experience pain the entire day." (A little; balance) | "My breast can be painful, but I don't experience pain continuously." (A little; balance) | Similarity [Female, 51 years, breast cancer] |
| [Range: Not at all - A little - Quite a bit - Very much] | "Normally I'm not in pain, but last Monday I was in a bit of pain because I was leaning forward while sitting in the car." (A little, balance) | "The past couple of weeks I experienced a lot of pain while eating. But the last few days the pain has diminished." (Very much; negative) | Dissimilarity [Male, 75 years, oesophageal cancer] |
| **Item 3 - Were you tired?** | "I sleep tight, and when I rise in the morning I feel fit. Until noon, I am able to do the housekeeping, and my concentration is at best. But from then on I get really tired." (Very much; negative) | "I can do the laundry, but I am not able to do other household chores." (Very much; negative) | Similarity [Male, 58 years, prostatic cancer] |
| [Range: Not at all - A little - Quite a bit - Very much] | "I was tired, but that was because of the narcosis. I haven't been tired after the anaestatic has worn off." (A little; balance) | "I got tired of having to come to the hospital every day, but I didn't get tired of the radiotherapy itself." (Not at all; positive) | Dissimilarity [Female, 49 years, breast cancer] |
| **Item 4 - Did you worry?** | "Up till now, I don't feel anything, I don't know anything, and I don't suffer from anything. But the doctor told me not to take it too light-heartedly. So I think I worry." (Quite a bit; balance) | "Actually, I don't worry. I won't be around to see it. But they say it is quite something, so I'll probably start to worry when I get the outcome of treatment." (A little; balance) | Similarity [Male, 78 years, oesophageal cancer] |
| [Range: Not at all - A little - Quite a bit - Very much] | "I have to overcome certain things, and I am limited in a number of things, but everything is within bounds." (Not at all; positive) | "I do worry, but not in the weekends, because I'm not getting treatment then and there's no need to make sure I have a filled bladder." (A little; balance) | Dissimilarity [Male, 66 years, prostatic cancer] |
| **Item 5 - Has your physical condition or medical treatment interfered with your social activities?** | "At the moment I'm on sick leave. I really enjoy my wok, I have friends there. (…) But I also have my social contacts at home. Now there are other people I can visit, people I don't get around to normally." (Not at all; positive) | "Normally I have my social contacts at work. At the moment I don't have these contacts, so I visited other people in order to retain my social contacts." (Not at all; positive) | Similarity [Male, 49 years, oesophageal cancer] |
| [Range: Not at all - A little - Quite a bit - Very much] | "I don't feel limited in my daily routine. But what troubles me is that I am no longer able to make plans." (Very much, negative) | "I can't plan anything, which I consider to be a limitation. However, I don't experience limitations in my daily routine." (Not at all; positive) | Dissimilarity [Female, 64 years, colorectal cancer] |
| **Item 6 - How would you rate your overall health during the past week?** | "Despite the lack of appetite in the past week, I consider my health as good." (6; positive) | "I was very tired, but my overall health was good." (6; positive) | Similarity [Female, 54 years, breast cancer] |
| [Range: 1 (very poor) - 7 (excellent)] | "There is only one small impairment. After the growth has been removed, I'll be completely fine again." (6; positive) | "[My health] isn't excellent, nor very poor." (4; balance) | Dissimilarity [Male, 57 years, oesophageal cancer] |
| **Item 7 - How would you rate your overall quality of life during the past week?** | "Not excellent, since I'm not completely recovered from surgery yet. However [my quality of life] is good." (6; positive) | "It isn't excellent, because of the diarrhea. But I consider my quality of life as good." (6; positive) | Similarity [Male, 64 years, prostatic cancer] |
| [Range: 1 (very poor) - 7 (excellent)] | "I always envisioned a future for myself. I don't have depressive traits at all, rather I am very optimistic. However, my quality of life during the past year was very poor. (…) At the moment I would say it's in the middle, it can get both worse and better." (4; balance) | "Physically, I wasn't able to do a thing, which restricted me to a great extent. (…) I consider my quality of life positively, because of my emotional state. I am happy with myself, and I have accepted this situation, which makes [my quality of life] very satisfying." (5; positive) | Dissimilarity [Female, 46 years, gynaecological cancer] |
| **Reporting and response selection**  **- (dis)similarity in the way a patient arrives at an answer and chooses the selected response category -** | **Baseline** | **Follow-up** | **(Dis)similarity** |
| **Item 1 - Do you have any trouble taking a short walk outside of the house?** | "I can walk to the rubbish bin, that's just around the corner. I do have trouble with it, but I am still able to walk outside." (Quite a bit) | "I can walk from here to the corner. I can walk, but don't ask how." (Quite a bit) | Similarity [Female, 76 years, bladder cancer] |
| [Range: Not at all - A little - Quite a bit - Very much] | "I can circle the answer 'very much' immediately. That's because my hip and knee no longer function as in the old days." (Very much) | "Quite a bit, that's because I'm not only short of breath, but I also have a painful hip and knee. (…) I would choose the answer 'very much' if I wasn't able to walk at all." (Quite a bit) | Dissimilarity [Male, 79 years, lung cancer] |
| **Item 2 - Have you had pain?** | "I experienced a little pain after surgery. But I have a high pain threshold. 'Not at all'…I'll have to take 'a little' then." ( A little) | "No, I haven't experienced any pain." (Not at all) | Similarity [Female, 50 years, breast cancer] |
| [Range: Not at all - A little - Quite a bit - Very much] | "I chose 'a little' because that's the first response option I saw. I could have picked 'quite a bit' just as well. Whether it is 'a little' or 'quite a bit' doesn't matter actually, it's both unsatisfactory." (A little) | "I have experienced pain when going to the bathroom. But it wasn't like the pain you have when you visit the dentist. Then I would go for 'very much' pain, but it wasn't that bad." (Quite a bit) | Dissimilarity [Male, 78 years, prostatic cancer] |
| **Item 3 - Were you tired?** | "I am tired 'quite a bit', and not 'very much'. Very much' is to feel completely exhausted." (Quite a bit) | "Very much, because I'm completely exhausted." (Very much) | Similarity [Male, 64 years, bladder cancer] |
| [Range: Not at all - A little - Quite a bit - Very much] | "It's not my nature to say I'm tired 'very much'." (Quite a bit) | "In the beginning, I wasn't tired due to radiation treatment at all. But the fatigue increased gradually." (Quite a bit) | Dissimilarity [Female, 64 years, colorectal cancer] |
| **Item 4 - Did you worry?** | "It's useless to worry." (A little) | "There's no point in worrying, because there's nothing you can do about it." (A little) | Similarity [Male, 64 years, prostatic cancer] |
| [Range: Not at all - A little - Quite a bit - Very much] | "I did worry a little, it is a surgery after all. But I've never had the feeling that my end was near." (A little) | "I worried a little about radiation treatment, but I worry a lot more about the upcoming chemotherapy. (…) To be honest, I worry 'quite a bit', but I don't want to admit it." (A little) | Dissimilarity [Male, 73 years, lung cancer] |
| **Item 5 - Has your physical condition or medical treatment interfered with your social activities?** | "Six months ago I quit my job due to my disease. At the moment I do some voluntary work, because I don't like to stay at home all day." (Quite a bit) | "Very much, because I can't do any work." (Very much) | Similarity [Male, 52 years, lung cancer] |
| [Range: Not at all - A little - Quite a bit - Very much] | "I cannot say I'm not limited at all, of course my body hinders me a little." (A little) | "Naturally, I am limited. I couldn't go shopping, I barely can walk and I can't visit my friends. So, 'very much'. No, I opt for 'quite a bit', I feel 'very much' doesn't suit me." (Quite a bit) | Dissimilarity [Female, 66 years, gynaecological cancer] |
| **Item 6 - How would you rate your overall health during the past week?** | "It isn't 'excellent', nor 'very poor'. It's average." (4) | "It isn't 'excellent', but it isn't very poor either. In between." (4) | Similarity [Female, 57 years, oesophageal cancer] |
| [Range: 1 (very poor) - 7 (excellent)] | "I think I should say a '5', but I circle the '6' because that's the direction I'm heading for." (6) | "[My health] isn't excellent, and a '6' is too close to excellent. So I would say a '5'." (5) | Dissimilarity [Male, 64 years, colorectal cancer] |
| **Item 7 - How would you rate your overall quality of life during the past week?** | "I separate quality of life from health. Since I just rated my overall health with a '5', I give my quality of life a '6'. (6) | "I don't rate my quality of life with the '4' I just rated my health with. This is different, quality of life encompasses more than health. So I go for a '5'. (5) | Similarity [Male, 58 years, prostatic cancer] |
| [Range: 1 (very poor) - 7 (excellent)] | "I would say a '7'. I rate my quality of life a few scores less than a '10' because of this limitation I now have." (7) | "It isn't 'very poor', rather moderate." (4) | Dissimilarity [Male, 67 years, oesophageal cancer] |

^1^ The interview excerpts are not exclusively related to one of the five cognitive processes, but might also be applicable in illustrating (dis)similarity in one or more of the remaining cognitive processes
